# The Association of Dietary Fiber Intake with Cardiometabolic Risk in Four Countries across the Epidemiologic Transition

**DOI:** 10.3390/nu10050628

**Published:** 2018-05-16

**Authors:** Louise Lie, Laquita Brown, Terrence E. Forrester, Jacob Plange-Rhule, Pascal Bovet, Estelle V. Lambert, Brian T. Layden, Amy Luke, Lara R. Dugas

**Affiliations:** 1 Public Health Sciences, Stritch School of Medicine, Maywood, IL 60153, USA; llie@luc.edu (L.L.); laquitab02@gmail.com (L.B.); Aluke@luc.edu (A.L.); 2 Solutions for Developing Countries, University of West Indies, Mona, Kingston, Jamaica; terrence.forrester@uwimona.edu.jm; 3 Department of Physiology, Kwame Nkrumah University of Science and Technology, Kumasi, Ghana; jprhule@gmail.com; 4 Unit for the Prevention and Control of Cardiovascular Disease, Ministry of Health, Victoria, Mahè Island, Republic of Seychelles; bovet.pascal@gmail.com; 5 Institute of Social and Preventive Medicine, University of Lausanne, Lausanne 1010, Switzerland; 6 Division for Exercise Science and Sports Medicine, Department of Human Biology, University of Cape Town, Cape Town 7700, South Africa; Vicki.Lambert@uct.ac.za; 7 Division of Endocrinology, Diabetes and Metabolism, University of Illinois at Chicago, Chicago, IL 60612, USA; blayde1@uic.edu; 8 Section of Endocrinology, Department of Medicine, Jesse Brown VA Medical Center, Chicago, IL 60612, USA

**Keywords:** cardiometabolic risk, dietary fiber, metabolic syndrome, obesity, epidemiologic transition

## Abstract

The greatest burden of cardiovascular disease is now carried by developing countries with cardiometabolic conditions such as metabolic syndrome, obesity and inflammation believed to be the driving force behind this epidemic. Dietary fiber is known to have protective effects against obesity, type 2 diabetes, cardiovascular disease and the metabolic syndrome. Considering the emerging prevalence of these cardiometabolic disease states across the epidemiologic transition, the objective of this study is to explore these associations of dietary fiber with cardiometabolic risk factors in four countries across the epidemiologic transition. We examined population-based samples of men and women, aged 25–45 of African origin from Ghana, Jamaica, the Seychelles and the USA. Ghanaians had the lowest prevalence of obesity (10%), while Jamaicans had the lowest prevalence of metabolic syndrome (5%) across all the sites. Participants from the US presented with the highest prevalence of obesity (52%), and metabolic syndrome (22%). Overall, the Ghanaians consumed the highest dietary fiber (24.9 ± 9.7 g), followed by Jamaica (16.0 ± 8.3 g), the Seychelles (13.6 ± 7.2 g) and the lowest in the USA (14.2 ± 7.1 g). Consequently, 43% of Ghanaians met the fiber dietary guidelines (14 g/1000 kcal/day), 9% of Jamaicans, 6% of Seychellois, and only 3% of US adults. Across all sites, cardiometabolic risk (metabolic syndrome, inflammation and obesity) was inversely associated with dietary fiber intake, such that the prevalence of metabolic syndrome was 13% for those in the lowest quartile of fiber intake, compared to 9% those in the highest quartile of fiber intake. Notably, twice as many of participants (38%) in the lowest quartile were obese compared to those in the highest quartile of fiber intake (18%). These findings further support the need to incorporate strategies and policies to promote increased dietary fiber intake as one component for the prevention of cardiometabolic risk in all countries spanning the epidemiologic transition.

## 1. Introduction

The high prevalence of cardiometabolic disease, including cardiovascular disease (CVD), metabolic syndrome, obesity and type 2 diabetes, has been well documented in developed countries [1,2,3]. These non-communicable diseases are increasing in prevalence in developing countries with rates approaching those of developed countries [4,5,6]. Here, we discuss these diseases as they relate to inflammation, obesity, and metabolic syndrome, a cluster of risk factors that elevate the risk of future CVD and includes raised fasting plasma glucose, abdominal obesity, and blood pressure [7]. The co-occurrence of these CVD risk factors characteristic of metabolic syndrome is believed to be the impetus driving the increasing CVD global prevalence.

Chronic low-grade inflammation is a pathological feature of chronic conditions including type 2 diabetes, metabolic syndrome, CVD and non-alcoholic fatty liver disease (NAFLD) [8,9]. Similarly, the key inflammatory marker, C-reactive protein (CRP), has been linked to insulin resistance, hypertension, development of type 2 diabetes and metabolic syndrome [10,11,12]. Chronic inflammation has been associated with poor lifestyle behavior such as dietary habits [13]. Diets high in dietary fiber (>30 g/day) have been shown to reduce levels of CRP [14], while inflammation characterized by serum levels of between 1 and 10 mg/L are associated with an increased risk of the development of CVD [15]. Notably, CRP has been well described as an independent risk marker for CVD [16].

Globally, as many as 75% of deaths due to coronary heart disease occur in developing countries [17]. The increasing prevalence of coronary heart disease in developing countries may be in part due to the epidemiologic transition [18], which describes the shift towards an industrialized consumer society, environmental, social and structural changes occur that may result in changes in morbidity and mortality patterns. In fact, over the last two decades, there has been a transition in the leading cause of death in developed countries from infectious etiologies to CVD and cancer [19]. Adverse lifestyle changes, such as increases in body weight, blood pressure, triglycerides, cholesterol levels, and diabetes prevalence have all been shown to contribute to increased exposure to CVD risk [20,21]. Notably, this transition has been associated with nutrition changes, due to the globalization of food production leading to a shift from traditional low fat, complex carbohydrate rich diets to the increased consumption of energy-dense, micronutrient and fiber poor foods [21].

Increasing dietary fiber intake may be one component of a multi-faceted approach to prevent cardiometabolic disease. Previous studies have found fiber to have protective effects against these related chronic health conditions, such as obesity [22], type 2 diabetes [23], cardiovascular disease [24] and metabolic syndrome [25,26]. Therefore, the objectives of this study are to examine the associations between dietary fiber and metabolic syndrome, inflammation and obesity in four countries of African origin spanning the epidemiologic transition.

## 2. Materials and Methods

### 2.1. Study Population and Ethics Approval

Initially, 2500 participants of African-descent aged 25–45 years from each of five countries: Ghana, Jamaica, South Africa, the Seychelles and the United States were enrolled in the Modelling the Epidemiologic Transition Study (METS) [27]. Briefly, five hundred participants, approximately 50% of whom are female, were enrolled in each of five study sites: rural Ghana, peri-urban South Africa, mixed urban/rural Seychelles, urban Jamaica and metropolitan Chicago. All participants, with the exception of the Seychelles, were of African descent. The five sites represented a range of social and economic development as defined by the UN Human Development Index (HDI) 2010; a rural Nkwantakese site in Ghana defined as a low HDI, and urban South African site as middle HDI and urban Seychelles and Jamaica as high HDI and a suburban Chicago site in the United States as a very high HDI country [28]. For the current analysis, however, due to concerns in the quality of dietary data from South Africa, this site was dropped. Baseline measures were conducted between January 2010 and December 2011. A detailed description of recruitment, measures and protocols has been previously published [27]. Approximately 50% of participants were female. The participant pool provided a range of body sizes with BMIs of participants from Ghana at a low of 24 kg/m^2^ to a high of 31 kg/m^2^ from the USA.

Persons with obvious infectious disease, pregnant or lactating women and HIV positive individuals, as well as individuals with conditions preventing them from engaging in normal physical activity such as severe arthritis and lower extremity disability, were excluded from the study.

The protocol for METS was approved by the Institutional Review Board of Loyola University Chicago, IL, USA (LU#200038); the Committee on Human Research Publication and Ethics of Kwame Nkrumah University of Science and Technology, Kumasi, Ghana; the Research Ethics Committee of the University of Cape Town, South Africa; the Board for Ethics and Clinical Research of the University of Lausanne, Switzerland; and the Ethics Committee of the University of the West Indies, Kingston, Jamaica; Written informed consent was obtained from all participants.

### 2.2. Dietary Intake

Each study participant completed two 24-h recalls using the multiple pass method [29,30,31], the first at the initial baseline visit and the second approximately 1 week later. In addition, 24-h recalls were recorded manually by trained interviewers and later entered electronically at the coordinating center at Loyola University Chicago. Dietary analysis was performed using the Nutrient Data System for Research (NDSR; University of Minneapolis, MN, USA) [29,30,31]. Food ingredient identification and portion size estimation were approximated from photographs of local foods at each site following methodology developed by the Medical Research Council South Africa [32]. The primary endpoint measures of interest were total energy intake and macronutrient composition. We also estimated the number of participants within each site meeting the Institute of Medicine recommendation that adults consume 14 g fiber/1000 kcal [33].

### 2.3. Biochemical Measures

Participants were asked to fast from the evening prior to the baseline clinic examination. Fasting blood samples were drawn for analysis of adipose-related hormones and adipocytokines, glucose, insulin, lipids, and albumin. The blood samples were processed and plasma or serum separated within two hours of collection and stored at −80 °C in the laboratory at each study site. Fasting plasma glucose was measured using the glucose oxidase method at each site at the time of collection. Insulin, total ghrelin, leptin and adiponectin from all sites were measured using radioimmunoassay kits (Linco Research, Inc., St. Charles, MO, USA) at the departmental laboratory at Loyola University Chicago. All remaining assays were conducted at the Zentrum fϋr Lambormedizin, Leiter Klinische Chemie und Hämatologie, St. Gallen, Switzerland.

### 2.4. Physical Activity Measurement

Physical activity (PA) was assessed using Actical accelerometers (Phillips Respironics, Bend, OR, USA) as previously described [34,35]. The Actical monitor was worn over an 8-day period, including during sleep. Raw data downloaded were first passed through a SAS) macro program [36] designed to infer non-wear time from 90 or more minutes of continuous zero activity counts. A valid day of PA monitoring was defined as one having 10 or more hours of wear time and participant files were included if they contained ≥4 or more valid days. Using the same protocol employed by the National Center for Health Statistics for the analysis of accelerometry data in the continuous National Health and Nutrition Examination Survey [37], minutes defined as comprising moderate, vigorous or moderate plus vigorous activity are presented as the total time in minutes (min) per day.

### 2.5. Clinical Outcomes

Metabolic syndrome was defined according to the Adult Treatment Panel III criteria [38,39] where individuals with at least three of the following cardiometabolic components were classified as having metabolic syndrome:

1. Waist circumference >102 cm in males and >88 cm in females;
2. Elevated blood pressure (≥130/85 mm Hg), or receiving treatment;
3. Hypertriglyceridemia (≥150 mg/dL), or receiving treatment;
4. Low high-density lipoprotein (HDL) cholesterol (<40 mg/dL in males and <50 mg/dL in females), or receiving treatment; and
5. Elevated fasting plasma glucose (≥100 mg/dL), or receiving treatment.

Inflammation was defined as C-reactive protein concentrations >3.0 mg/L [40,41]. Individuals with a body mass index ≥30 kg/m^2^ were defined as obese [42].

### 2.6. Statistical Analyses

Participant characteristics and cardiometabolic risk factors were summarized using means ± standard deviations (SD), after being examined for normality. Proportions were calculated and presented as *n* (%) for dichotomous variables. Fiber quartiles were determined for each site as well as across all sites based on mean individual dietary fiber intake (g/day). Comparison of cardiometabolic risks by fiber quartiles at each site was performed by Pearson’s chi-squared test with statistical significance noted for *p ≤* 0.05. Multi-variable logistic regression was performed with the outcomes of metabolic syndrome, inflammation and obesity stratified by dietary fiber quartile after adjusting for age, gender, energy intake and smoking. Statistical analyses were performed using Stata (version 12, Manufacturer, College Station, TX, USA).

## 3. Results

Data from a total of 1940 participants from four sites were included in this analysis. Descriptive characteristics of participants, by site, are presented in Table 1. The mean age of participants was similar across all sites ranging from 34.2 ± 6.7 years in Ghana to 36.2 ± 5.6 years in Seychelles. Weight, height and subsequent body mass index varied greatly across all sites with Ghanaians being shorter (162.6 ± 8.2 cm), lighter (63.5 ± 11.5 kg) and having lower body mass indices (24.1 ± 4.5 kg/m^2^) compared to all other sites. Conversely, US participants were the tallest (169.9 ± 9.0 cm), heaviest (91.9 ± 24.2 kg) and had the highest body mass indices (31.9 ± 8.4 kg/m^2^) across all the sites. Waist circumference measures followed the same trend with Ghana on the lowest end of the range (81.2 ± 12.1 cm) and US participants on the highest end (99.6 ± 20.4 cm). Similarly, systolic and diastolic blood pressures were the lowest in Ghana (113.90 ± 14.8 mmHg and 67.1 ± 11.3 mmHg, respectively) and highest in the USA (122.6 ± 16.3 mmHg and 80.5 ± 12.8 mmHg, respectively).

The US participants had the highest mean values of all biochemical measures of cardiometabolic risk factors (total cholesterol, HDL-C, triglycerides, blood glucose and CRP). The lowest mean values of biochemical measures of cardiometabolic risk factors were distributed amongst the lower and mid HDI countries with lowest total cholesterol and HDL-C values observed in Ghana, triglycerides and blood glucose in Jamaica and CRP in Seychelles.

The highest proportion of smokers were from the US (46.3%) and the lowest proportion of smokers were from Ghana (2.7%). Participants from the US accumulated the lowest amount of daily moderate-to-vigorous PA (23.2 ± 28.9 min/day) compared to the Ghanaians who spent more than 30 mins/day in moderate-to-vigorous activity.

Habitual dietary intake data are summarized in Table 2. The US participants consumed a diet that was the most energy rich (2294 ± 889.3 kcal/day) compared to the three sites. They also consumed the lowest amount of total dietary fiber (14.2 ± 7.1 g/day), and insoluble dietary fiber (9.5 ± 5.4 g/day). Conversely, Ghanaians consumed the highest dietary fiber (24.9 ± 9.7 g/day) and insoluble dietary fiber (18.8 ± 7.5 g/day). Overall, 43% of Ghanaians met the Institute of Medicine (IOM) fiber guideline [>14 g fiber/1000 kcal/day, [33]], compared to only 9% of Jamaicans, 6% of participants from Seychelles, and only 3% of participants from the US. Overall, the Ghanaian diet was low in fat and protein and high in the proportion of carbohydrates, with 21.6% of their energy coming from fat, 11.9% from protein and 65.9% from carbohydrates. In contrast, the US diet was high in fat and protein and low in carbohydrates with 36.6% of their energy from fat, 15.6% from protein and 45.8% from carbohydrates. Interestingly, protein intake (18.4 ± 4.7% energy intake) was highest among participants from Seychelles, reflecting the large contribution of seafood from this island nation.

### 3.1. Cardiometabolic Risk Factors across the Epidemiologic Transition

**Figure 1:**
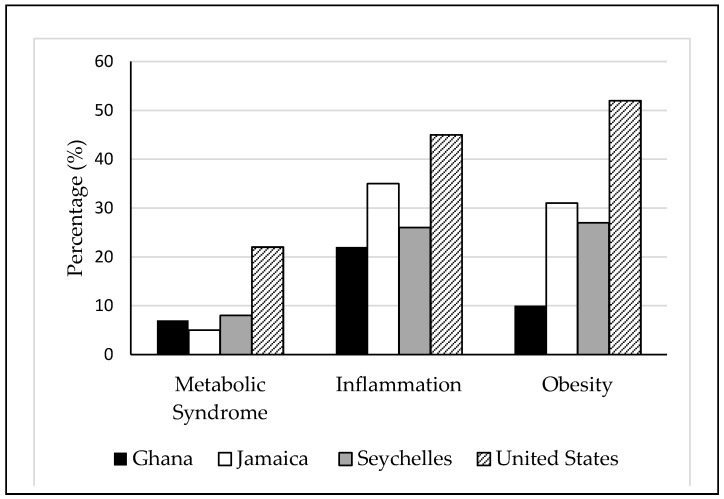
Comparison of prevalence of cardiometabolic risk by site. Metabolic syndrome was defined according to the Adult Treatment Panel III criteria [38,39] where individuals had at least three cardiometabolic components. Inflammation was defined as C-reactive protein concentrations >3.0 mg/L [40,41]. Individuals with a body mass index ≥30 kg/m^2^ were defined as obese [42].

The US participants consistently presented with the greatest prevalence of cardiometabolic risk factors, including metabolic syndrome (22%), inflammation (45%) and obesity (52%). While Jamaicans had the lowest levels of metabolic syndrome (5%), they presented with unexpectedly high levels of inflammation (35%) and obesity (31%). Ghanaians presented with the lowest proportion of inflammation (22%) and obesity (10%). A comparison of cardiometabolic risk by site can be seen in Figure 1.

### 3.2. The Association of Dietary Fiber Intake on Cardiometabolic Risk Factors across the Epidemiologic Transition

The prevalence of cardiometabolic risk factors was analyzed by chi-square analysis comparing the lowest fiber quartile (Q1) to the highest fiber quartile (Q4) specific to each site to determine the effect of dietary fiber intake on cardiometabolic risk at each site. The trend for the lower prevalence of cardiometabolic risk factors with increased dietary fiber was seen for most cardiometabolic conditions in most sites, apart from inflammation in Ghana and Jamaica and metabolic syndrome in the Seychelles. However, it should be noted, that differences in the prevalence of the cardiometabolic risk factors and fiber quartiles were modest with statistical significance achieved only in the prevalence of inflammation in the Seychelles and the USA and metabolic syndrome in the USA.

Mean dietary fiber intake across all sites was 17.3 g/day. The comparison of cardiometabolic risk factors by dietary fiber quartiles across all sites followed the trend seen by inter-site comparison. Overall, cardiometabolic risk decreased with increasing dietary fiber intake. For example, the prevalence of metabolic syndrome in participants from the lowest quartile was 12.5% compared to the 9.0% in the highest quartile (*p* = 0.089), as seen in Table 3. Similarly, 38.0% of participants from the lowest quartile presented with increased inflammatory CRP, compared to 23.8% of participants in the highest quartile (*p* < 0.001). Finally, approximately 38.2% of participants in the lowest quartile of fiber intake were obese, compared to 17.6% in the highest quartile of fiber intake (*p* < 0.001).

In Table 4, the adjusted multivariate logistic regression models demonstrate an inverse relationship between dietary fiber intake and cardiometabolic risk. After adjusting for age, gender, energy intake, PA and smoking, increasing fiber intake is associated with protection from the metabolic syndrome. Specifically, participants who consumed more than 22.1 g/day of fiber were 29% less likely to present with metabolic syndrome, compared to participants consuming less than 10.3 g/day of dietary fiber. Similarly, people consuming greater than 22.1 g/day of fiber were 47% less likely to have elevated CRP values, compared to those only consuming less than 10.3 g/day of dietary fiber. Finally, participants consuming more than 22.1 g/day were 66% less likely to be obese, compared to those consuming less than 10.3 g/day of dietary fiber.

## 4. Discussion

This study investigated the association of cardiometabolic risk factors and dietary fiber intake in four diverse populations spanning the epidemiologic transition. The US is considered a very high HDI designation, and the self-reported diets consist largely of processed and fast foods that are energy dense, of low nutritional value and very low in fiber content. The data shown here illustrate the nutritional transition across the epidemiologic transition [43,44]. Historically, the rapid modernization seen during the formation of the People’s Republic of China resulted in dramatic changes to diet, physical activity and obesity-related changes similar to that seen in developing countries [44]. One such diet change was the transition towards refined grains and away from high fiber containing coarser grains that involved a shift from traditional diets to processed high-fat and sugary foods. Similar results can be seen in developing countries [44,45,46,47].

The 2015 Dietary guidelines for Americans recommend that females 31–50 years of age is 25.2 g/day and 30.8 g/day for males of the same age [48], while the Institute of Medicine recommends that adults consume >14 g fiber/1000 kcal [33]. The data presented here show that dietary fiber intake is well below recommended amounts and this is particularly evident in the US participants, where mean dietary fiber intake was 14.2 ± 7.1 g/day, with only 3% of the study population meeting the Institute of Medicine fiber intake guideline. This is in line with previously reported USA estimates for dietary fiber intake of 15.7–17.0 g/day [49] for non-Hispanic blacks, who only consumed about 14 g/day, well below the recommended daily fiber intake. The Ghanaian diet had the highest dietary fiber intake (24.9 ± 9.7 g/day) due to fiber rich foods like cassava (eaten as banku or fufu) eaten as staples with most meals in rural Ghanaian villages. Despite this, only 43% of Ghanaians met the Institute of Medicine fiber intake guideline of >14 g fiber/1000 kcal.

In our study, we found a heterogeneous pattern of cardiometabolic risk factors in the four populations spanning the epidemiologic transition. Collectively, the prevalence of cardiometabolic risk factors are lowest in Ghana (lowest HDI) and highest in the USA (highest HDI) compared to the other sites analyzed in this study. When comparing the prevalence of cardiometabolic risk factors between participants consuming either the lowest or highest daily dietary fiber across all sites, a significant association is seen for higher inflammation, indicated by elevated CRP levels and obesity in the participants with the lowest daily fiber intake. Although no statistical significance was reached between indicators of metabolic syndrome and dietary fiber quartiles, a similar trend was observed. Similarly, non-significant trends were observed within each site. This could be due to sampling methods where intra-site study participants were geographically very close (within the same communities or villages) and therefore represented very similar lifestyle exposures and risk factors. This resulted in little variability within the site. However, overall, the findings from this study corroborate with many other studies, finding that dietary fiber may have a protective effect against cardiometabolic risk [22,23,24,25,26]. In our study, participants with the highest daily dietary fiber intakes had significantly lower odds for cardiometabolic risk, after adjusting for other known lifestyle risk factors. Notably, we have shown that consuming more dietary fiber is associated with a decreased odds of cardiometabolic risk, and that the odds of metabolic syndrome, inflammation and obesity decrease with a higher fiber intake (>22 g), after accounting for the effects age, gender, smoking, low PA, and energy intake (Table 4). The implications of this are that concerted efforts should be made to increase fiber intake, and that following the recommended amount is optimal for cardiometabolic risk reduction [48].

This study represents one of a few studies examining cardiometabolic risk across multiple sites spanning the epidemiological transition, particularly in people of African origin. Standardized questionnaires, protocols, analysis and methodology allow us to make these comparisons across all sites. However, we recognize limitations to our study. Firstly, while dietary fiber intake is the main focus of the current paper, it is likely only one modifiable component of a multi-faceted approach to prevent and treat cardiometabolic risk. Other important dietary aspects may also contribute to cardiometabolic risk [50]. For example, the US participants not only consumed the lowest daily amount of fiber, but they also consumed the greatest number of calories, and had the greatest proportion of energy from fat. In addition, they also accumulated the least amount of moderate-to-vigorous daily PA, and had the most smokers. Taken together, it is therefore not surprising that the US cohort presented with the greatest cardiometabolic disease prevalence. However, these other factors were adjusted for in the analysis, and dietary fiber intake remained significantly inversely associated with cardiometabolic outcomes. Secondly, within in each site, the sample size was relatively small, and not necessarily representative of that country as a whole. Thirdly, the study does not include the elderly who are at increased risk of cardiometabolic disease. However, the narrow range used in this study allows for direct comparisons across sites. Fourth, dietary information was dependent on self-report on 24 h recall. It is well known that this frequently results in underestimation of portion size and recall bias. Finally, this study focused only on total dietary fiber; however, the type of dietary fiber may be important to its association with cardiometabolic risk.

## 5. Conclusions

This study has shown that dietary fiber intake varies across the epidemiologic transition, and is inversely associated with cardiometabolic risk factors, which similarly range from the least prevalent in the lower HDI counties (Ghana) to the greatest prevalence in higher HDI country (US). The significant association between these risk factors and dietary fiber intake suggest that this relationship may be dose dependent. These findings further support the need to incorporate strategies and policies to promote increased dietary fiber intake as one component for the prevention of cardiometabolic risk in all countries spanning the epidemiologic transition.

## Figures and Tables

**Table 1:** Descriptive characteristics of study population by site.

	Ghana	Jamaica	Seychelles	United States
	*N* = 489	*N* = 402	*N* = 484	*N* = 444
**Demographics**				
Female (*n*, %)	287 (58.7)	244 (60.7)	261 (53.9)	228 (51.4)
Age (year)	34.2 ± 6.8	34.4 ± 6.1	36.2 ± 5.6	35.2 ± 6.3
**Anthropometrics**				
Weight (kg)	63.5 ± 11.5	75.9 ± 17.1	75.8 ± 17.0	91.9 ± 24.2
Height (cm)	162.6 ± 8.2	169.6 ± 9.2	167.1 ± 8.8	169.9 ± 9.0
Body Mass Index (kg/m^2^)	24.1 ± 4.5	26.6 ± 6.4	27.1 ± 5.6	31.9 ± 8.4
Waist Circumference (cm)	81.2 ± 12.1	86.1 ± 14.2	88.7 ± 12.0	99.6 ± 20.4
Systolic Blood Pressure (mmHg)	113.9 ± 14.8	118.4 ± 14.2	116.2 ± 14.7	122.6 ± 16.3
Diastolic Blood Pressure (mmHg)	67.1 ± 11.3	71.7 ± 11.3	73.0 ± 10.7	80.5 ± 12.8
**Biochemical Measures**				
Cholesterol (mg/dL)	161.4 ± 35.3	162.8 ± 33.9	170.9 ± 35.4	181.1 ± 38.4
HDL-C (mg/dL) Ratio total cholesterol to HDL	46.0 ± 14.4 3.8 ± 1.7	46.5 ± 12.1 3.7 ± 1.0	47.7 ± 12.8 3.9 ± 1.2	50.8 ± 14.6 3.8 ± 1.1
Triglycerides (mg/dL)	82.0 ± 40.3	73.2 ± 36.3	79.8 ± 60.6	97.3 ± 57.4
Blood glucose (mg/dL)	100.3 ± 12.2	93.1 ± 9.4	100.9 ± 29.5	103.5 ± 35.5
CRP (mg/dL)	4.7 ± 13.3	4.2 ± 6.3	3.1 ± 4.5	6.0 ± 11.0
**Lifestyle Habits**				
Smoker or ex-smoker (*n*, %) Moderate-to-vigorous PA (min/day)	13 (2.8) 34.3 ± 22.6	101 (25.1) 23.3 ± 19.3	95 (19.6) 28.9 ± 20.9	205 (46.3) 23.3 ± 28.9

**Table 2:** Dietary analysis of study population by site.

	Ghana	Jamaica	Seychelles	United States
	*N* = 489	*N* = 402	*N* = 484	*N* = 444
Energy (kcal) % Energy from fat % Energy from saturated fat % Energy from monounsaturated fat % Energy from polyunsaturated fat	1849 ± 495.7 21.6 ± 9.2 7.1 ± 4.1 8.2 ± 3.7 4.6 ± 2.5	1930 ± 590.5 25.7 ± 6.6 9.4 ± 4.1 8.6 ± 2.6 5.5 ± 2.1	1844 ± 590.9 28.4 ± 7.7 8.4 ± 2.9 8.9 ± 3.0 8.6 ± 3.4	2294 ± 889.3 36.6 ± 7.0 11.8 ± 2.9 13.6 ± 3.1 7.9 ± 2.8
% Energy from carbohydrates % Energy from protein	65.9 ± 10.4 11.9 ± 4.0	58.6 ± 8.4 14.7 ± 3.9	51.3 ± 9.4 18.4 ± 4.7	45.8 ± 9.4 15.6 ± 4.1
Meeting 14 g/1000 kcal (*n*, %)	208 (42.5)	35 (8.7)	28 (5.8)	14 (3.2)
Dietary fiber (g) Soluble fiber (g) Insoluble dietary fiber (g)	24.9 ± 9.7 6.1 ± 2.8 18.8 ± 7.5	16.0 ± 8.3 4.8 ± 2.6 11.6 ± 6.1	13.6 ± 7.2 3.9 ± 2.2 9.6 ± 5.4	14.2 ± 7.1 4.6 ± 2.4 9.5 ± 5.4
Ratio soluble to insoluble dietary fiber	0.3 ± 0.1	0.4 ± 0.2	0.4 ± 0.2	0.6 ± 0.3
Meeting 14 g/1000 kcal (*n*, %)	208 (42.5)	35 (8.7)	28 (5.8)	14 (3.2)

**Table 3:** Comparison of cardiometabolic risks by fiber quartiles at each site, *N* (%).

	Ghana		Jamaica		Seychelles		USA		Across All Sites	
	*n* = 246		*n* = 200		*n* = 242		*n* = 222		*n* = 942	
	Q1	Q4 ^b^	Q1	Q4 ^c^	Q1	Q4 ^d^	Q1	Q4 ^e^	Q1	Q4 ^f^
Metabolic syndrome	9 (7.3)	8 (6.5)	10 (10.0)	6 (6.0)	8 (6.6)	12 (9.9)	30 (27.0)	15 (13.5) *	57 (12.5)	41 (9.0)
Inflammation	26 (23.0)	29 (24.8)	27 (30.3)	34 (36.6)	33 (31.1)	22 (19.5) *	51 (51.0)	39 (37.5) *	154 (38.0)	103 (23.8) *
Obesity	12 (9.8)	10 (8.1)	34 (34.0)	27 (27.0)	39 (32.2)	30 (24.8)	56 (50.5)	54 (48.7)	173 (38.0)	80 (17.6) *

* *p* ≤ 0.05; ^a^ Q1 represents the lowest quartile of fiber intake, and Q4, the highest. ^b^ Q1 < 17.84, Q4 > 30.88; ^c^ Q1 < 9.92, Q4 > 19.80 ^d^ Q1 < 8.99, Q4 > 17.18; ^e^ Q1 < 8.78, Q4 > 17.55; ^f^ Q1 < 10.34, Q4 > 22.10.

**Table 4:** Adjusted odds ratios for cardiometabolic risk factors, based on quartiles of dietary fiber intake in four countries across the epidemiologic transition ^1^.

Quartiles of Total Fiber	Metabolic Syndrome	Inflammation	Obesity
0.0–10.3 g (Q1)	1	1	1
10.3–15.1 g (Q2)	1.0 (0.7–1.5)	0.9 (0.7–1.3)	0.9 (0.7–1.2)
15.1–22.1 g (Q3)	0.5 (0.3–0.8) *	0.7 (0.5–0.9) *	0.6 (0.4–0.8) *
>22.1 g (Q4)	0.7 (0.4–1.2)	0.5 (0.4–0.8) *	0.4 (0.2–0.5) *

^1^ Multivariate model adjusted for age, gender, energy intake, physical activity and smoking. * *p* ≤ 0.05.

## References

[1] Benjamin E.J., Blaha M.J., Chiuve S.E., Cushman M., Das S.R., Deo R., de Ferranti S.D., Floyd J., Fornage M., Gillespie C. (2017). Heart Disease and Stroke Statistics—2017 Update: A Report From the American Heart Association. Circulation.

[2] Zimmet P.Z., Magliano D.J., Herman W.H., Shaw J.E. (2014). Diabetes: A 21st century challenge. Lancet Diabetes Endocrinol..

[3] Geiss L.S., Kirtland K., Lin J., Shrestha S., Thompson T., Albright A., Gregg E.W. (2017). Changes in diagnosed diabetes, obesity, and physical inactivity prevalence in US counties, 2004–2012. PLoS ONE.

[4] Durazo-Arvizu R.A., Luke A., Cooper R.S., Cao G., Dugas L., Adeyemo A., Adeyemo A., Boyne M., Forrester T. (2008). Rapid increases in obesity in Jamaica, compared to Nigeria and the United States. BMC Public Health.

[5] Eckert S., Kohler S. (2014). Urbanization and health in developing countries: A systematic review. World Health Popul..

[6] Ford N.D., Patel S.A., Narayan K.M. (2017). Obesity in Low- and Middle-Income Countries: Burden, Drivers, and Emerging Challenges. Annu. Rev. Public Health.

[7] George A., Zimmet P.Z., Shaw J.E., Grundy S.M. (2006). The IDF Consensus Worldwide Definition of the Metabolic Syndrome.

[8] Libby P. (2002). Inflammation in atherosclerosis. Nature.

[9] Hotamisligil G.S. (2006). Inflammation and metabolic disorders. Nature.

[10] Calder P.C., Albers R., Antoine J.M., Blum S., Bourdet-Sicard R., Ferns G.A., Folkerts G., Friedmann P.S., Frost G.S., Guarner F. (2009). Inflammatory disease processes and interactions with nutrition. Br. J. Nutr..

[11] Calder P.C., Ahluwalia N., Brouns F., Buetler T., Clement K., Cunningham K., Cunningham K., Esposito K., Jönsson L.S., Kolb H. (2011). Dietary factors and low-grade inflammation in relation to overweight and obesity. Br. J. Nutr..

[12] Calder P.C., Ahluwalia N., Albers R., Bosco N., Bourdet-Sicard R., Haller D., Holgate S.T., Jönsson L.S., Latulippe M.E., Marcos A. (2013). A consideration of biomarkers to be used for evaluation of inflammation in human nutritional studies. Br. J. Nutr..

[13] Suganami T., Tanimoto-Koyama K., Nishida J., Itoh M., Yuan X., Mizuarai S., Kotani H., Yamaoka S., Miyake K., Aoe S. (2007). Role of the Toll-like receptor 4/NF-kappaB pathway in saturated fatty acid-induced inflammatory changes in the interaction between adipocytes and macrophages. Arterioscler. Thromb. Vasc. Biol..

[14] King D.E., Egan B.M., Woolson R.F., Mainous A.G., Al-Solaiman Y., Jesri A. (2007). Effect of a high-fiber diet vs a fiber-supplemented diet on C-reactive protein level. Arch. Intern. Med..

[15] Kruger H.S. (2010). Associations of serum C-reactive protein with physical activity, fitness and fatness in South African adolescents. Cardiovasc. J. Afr..

[16] Ridker P.M., Buring J.E., Cook N.R., Rifai N. (2003). C-reactive protein, the metabolic syndrome, and risk of incident cardiovascular events: An 8-year follow-up of 14,719 initially healthy American women. Circulation.

[17] Alberti K.G., Zimmet P., Shaw J., Group IDFETFC (2005). The metabolic syndrome—A new worldwide definition. Lancet.

[18] Dugas L.R., Forrester T.E., Plange-Rhule J., Bovet P., Lambert E.V., Durazo-Arvizu R.A., Cao G., Cooper R.S., Khatib R., Tonino L. (2017). Cardiovascular risk status of Afro-origin populations across the spectrum of economic development: Findings from the Modeling the Epidemiologic Transition Study. BMC Public Health.

[19] WHO (2002). The World Health Report 2002: Reducing Risks, Promoting Healthy Life.

[20] Reddy K.S., Yusuf S. (1998). Emerging epidemic of cardiovascular disease in developing countries. Circulation.

[21] Gaziano T.A., Bitton A., Anand S., Abrahams-Gessel S., Murphy A. (2010). Growing epidemic of coronary heart disease in low- and middle-income countries. Curr. Probl. Cardiol..

[22] Lang T. (1998). The new globalisation, food and health: Is public health receiving its due emphasis?. J. Epidemiol. Commun. Health.

[23] Tucker L.A., Thomas K.S. (2009). Increasing total fiber intake reduces risk of weight and fat gains in women. J. Nutr..

[24] Montonen J., Knekt P., Jarvinen R., Aromaa A., Reunanen A. (2003). Whole-grain and fiber intake and the incidence of type 2 diabetes. Am. J. Clin. Nutr..

[25] Ludwig D.S., Pereira M.A., Kroenke C.H., Hilner J.E., Van Horn L., Slattery M.L. (1999). Dietary fiber, weight gain, and cardiovascular disease risk factors in young adults. JAMA.

[26] Galisteo M., Duarte J., Zarzuelo A. (2008). Effects of dietary fibers on disturbances clustered in the metabolic syndrome. J. Nutr. Biochem..

[27] Luke A., Bovet P., Forrester T.E., Lambert E.V., Plange-Rhule J., Schoeller D.A., Dugas L.R., Durazo-Arvizu R.A., Shoham D., Cooper R.S. (2012). Protocol for the modeling the epidemiologic transition study: A longitudinal observational study of energy balance and change in body weight, diabetes and cardiovascular disease risk. BMC Public Health.

[28] Barro R.J., Lee J.W. (2011). A New Data Set of Educational Attainment in the World, 1950–2010.

[29] Carriquiry A.L. (2003). Estimation of usual intake distributions of nutrients and foods. J. Nutr..

[30] Carriquiry A.L., Fuller W.A., Goyeneche J.J., Dodd K.W. (1995). Estimation of the Usual Intake Distributions of Ratios of Dietary Components.

[31] Guenther P.M., Kott P.S., Carriquiry A.L. (1997). Development of an approach for estimating usual nutrient intake distributions at the population level. J. Nutr..

[32] Steyn N.P., Nel J.H., Parker W.A., Ayah R., Mbithe D. (2011). Dietary, social, and environmental determinants of obesity in Kenyan women. Scand. J Public Health.

[33] Institute of Medicine (U.S.) Panel on Macronutrients. II. Institute of Medicine (U.S.). Standing Committee on the Scientific Evaluation of Dietary Reference Intakes. QP141.D529 2005.. https://www.nal.usda.gov/sites/default/files/fnic_uploads/energy_full_report.pdf.

[34] Luke A., Bovet P., Plange-Rhule J., Forrester T.E., Lambert E.V., Schoeller D.A., Dugas L.R., Durazo-Arvizu R.A., Shoham D.A., Cao G. (2014). A mixed ecologic-cohort comparison of physical activity & weight among young adults from five populations of African origin. BMC Public Health.

[35] Dugas L.R., Bovet P., Forrester T.E., Lambert E.V., Plange-Rhule J., Durazo-Arvizu R.A., Shoham D., Kroff J., Cao G., Cooper R.S. (2014). Comparisons of intensity-duration patterns of physical activity in the US, Jamaica and 3 African countries. BMC Public Health.

[36] (2014). SAS Programs for Analyzing NHANES 2003–2004 Accelerometer Data. http://appliedresearch.cancer.gov/nhanes_pam/.

[37] Troiano R.P., Berrigan D., Dodd K.W., Masse L.C., Tilert T., McDowell M. (2008). Physical activity in the United States measured by accelerometer. Med. Sci. Sports Exerc..

[38] National Cholesterol Education Program, Expert Panel on Detection, Evaluation, and Treatment of High Blood Cholesterol in Adults (2002). Third Report of the National Cholesterol Education Program (NCEP) Expert Panel on Detection, Evaluation, and Treatment of High Blood Cholesterol in Adults (Adult Treatment Panel III) final report. Circulation.

[39] Grundy S.M., Cleeman J.I., Daniels S.R., Donato K.A., Eckel R.H., Franklin B.A., Gordon D.J., Krauss R.M., Savage P.J., Smith S.C. (2005). Diagnosis and management of the metabolic syndrome: An American Heart Association/National Heart, Lung, and Blood Institute Scientific Statement. Circulation.

[40] Ford E.S., Giles W.H., Mokdad A.H., Myers G.L. (2004). Distribution and correlates of C-reactive protein concentrations among adult US women. Clin. Chem..

[41] Rifai N., Ridker P.M. (2003). Population distributions of C-reactive protein in apparently healthy men and women in the United States: Implication for clinical interpretation. Clin. Chem..

[42] Expert Panel on the Identification, Evaluation, and Treatment of Overweight in Adults (1998). Clinical guidelines on the identification, evaluation, and treatment of overweight and obesity in adults: Executive summary. Am. J. Clin. Nutr..

[43] Popkin B.M. (2014). Synthesis and implications: China’s nutrition transition in the context of changes across other low- and middle-income countries. Obes. Rev..

[44] Popkin B.M., Slining M.M. (2013). New dynamics in global obesity facing low- and middle-income countries. Obes. Rev..

[45] Popkin B.M., Adair L.S., Ng S.W. (2012). Global nutrition transition and the pandemic of obesity in developing countries. Nutr. Rev..

[46] Popkin B.M., Gordon-Larsen P. (2004). The nutrition transition: Worldwide obesity dynamics and their determinants. Int. J. Obes. Relat. Metab. Disord..

[47] Popkin B.M., Paeratakul S., Zhai F., Ge K. (1995). A review of dietary and environmental correlates of obesity with emphasis on developing countries. Obes. Res..

[48] U.S. Department of Health and Human Services, U.S. Department of Agriculture (2015). 2015–2020 Dietary Guidelines for Americans. 8th Edition. https://health.gov/dietaryguidelines/2015/guidelines/.

[49] Grooms K.N., Ommerborn M.J., Pham D.Q., Djousse L., Clark C.R. (2013). Dietary fiber intake and cardiometabolic risks among US adults, NHANES 1999–2010. Am. J. Med..

[50] Steyn N.P., McHiza Z.J. (2014). Obesity and the nutrition transition in Sub-Saharan Africa. Ann. N. Y. Acad. Sci..

